# Neurokernel: An Open Source Platform for Emulating the Fruit Fly Brain

**DOI:** 10.1371/journal.pone.0146581

**Published:** 2016-01-11

**Authors:** Lev E. Givon, Aurel A. Lazar

**Affiliations:** Department of Electrical Engineering, Columbia University, New York, NY 10027, United States of America; SUNY Downstate MC, UNITED STATES

## Abstract

We have developed an open software platform called Neurokernel for collaborative development of comprehensive models of the brain of the fruit fly *Drosophila melanogaster* and their execution and testing on multiple Graphics Processing Units (GPUs). Neurokernel provides a programming model that capitalizes upon the structural organization of the fly brain into a fixed number of functional modules to distinguish between these modules’ local information processing capabilities and the connectivity patterns that link them. By defining mandatory communication interfaces that specify how data is transmitted between models of each of these modules regardless of their internal design, Neurokernel explicitly enables multiple researchers to collaboratively model the fruit fly’s entire brain by integration of their independently developed models of its constituent processing units. We demonstrate the power of Neurokernel’s model integration by combining independently developed models of the retina and lamina neuropils in the fly’s visual system and by demonstrating their neuroinformation processing capability. We also illustrate Neurokernel’s ability to take advantage of direct GPU-to-GPU data transfers with benchmarks that demonstrate scaling of Neurokernel’s communication performance both over the number of interface ports exposed by an emulation’s constituent modules and the total number of modules comprised by an emulation.

## Introduction

Reverse engineering the information processing functions of the brain is an engineering grand challenge of immense interest that has the potential to drive important advances in computer architecture, artificial intelligence, and medicine. The human brain is an obvious and tantalizing target of this effort; however, its structural and architectural complexity place severe limitations upon the extent to which models built and executed with currently available computational technology can relate its biological structure to its information processing capabilities. Successful development of human brain models must therefore be preceded by an increased understanding of the structural/ architectural complexity of the more tractable brains of simpler organisms and how they implement specific information processing functions and govern behavior [1].

The nervous system of the fruit fly *Drosophila melanogaster* possesses a range of features that recommend it as a model organism of choice for relating brain structure to function. Despite the obvious differences in size and complexity between the mammalian and fruit fly brains, researchers dating back to Cajal have observed common design principles in the structure of their sensory subsystems [2]. Many of the genes and proteins expressed in the mammalian brain are also conserved in the genome of *Drosophila* [3]. These features strongly suggest that valuable insight into the workings of the mammalian brain can be obtained by focusing on that of *Drosophila*.

Remarkably, the fruit fly is capable of a host of complex nonreactive behaviors that are governed by a brain containing only ∼10^5^ neurons and ∼10^7^ synapses organized into fewer than 50 distinct functional units, many of which are known to be directly involved in functions such as sensory processing, locomotion, and control [4]. The relationship between the fruit fly’s brain and its behaviors can be experimentally probed using a powerful toolkit of genetic techniques for manipulation of the fruit fly’s neural circuitry such as the GAL4 driver system [5–9], recent advances in experimental methods for precise recordings of the fruit fly’s neuronal responses to stimuli [10–12], techniques for analyzing the fly’s behavioral responses to stimuli [13–15], and progress in reconstruction of the fly connectome, or neural connectivity map [16, 17]. These techniques have provided access to an immense amount of valuable structural and behavioral data that can be used to model how the fruit fly brain’s neural circuitry implements processing of sensory stimuli [4, 18–22].

Despite considerable progress in mapping the fruit fly’s connectome and elucidating the patterns of information flow in its brain, the complexity of the fly brain’s structure and the still-incomplete state of knowledge regarding its neural circuitry pose challenges that go beyond satisfying the current computational resource requirements of fly brain models. These include (1) the need to explicitly target the information processing capabilities of functional units in the fruit fly brain, (2) the need for fly brain model implementations to efficiently scale over additional hardware resources as they advance in complexity, and (3) the need for brain modeling to be approached as an explicitly open and collaborative process of iterative refinement by multiple parties similar to that successfully employed in the design of the Internet [23] and large open source projects such as the Python programming language [24].

To address these challenges, we have developed an open source platform called Neurokernel for implementing connectome-based fruit fly brain models and executing them upon multiple Graphics Processing Units (GPUs). In order to achieve scaling over multiple computational resources while providing the programmability required to model the constituent functional modules in the fly brain, the Neurokernel architecture provides features similar to that of an operating system kernel. In contrast to general-purpose neural simulators, the design of Neurokernel and brain models built upon it is driven by publicly available proposals called Requests for Comments (RFCs).

Neurokernel’s design is predicated upon the organization of the fruit fly brain into a fixed number of functional modules characterized by local neural circuitry. Neurokernel explicitly enforces a programming model for implementing models of these functional modules called Local Processing Units (LPUs) that separates between their internal design and the connectivity patterns that link their external communication interfaces independently of the internal design of models designed by other researchers and of the connectivity patterns that link them. This modular architecture facilitates collaboration between researchers focusing on different functional modules in the fly brain by enabling models independently developed by different researchers to be integrated into a single whole brain model irrespective of their internal designs.

This paper is organized as follows. We first review the anatomy of the fruit fly brain that motivate Neurokernel’s design and then describe its architecture and support for GPU resources and programmability in the following section. The subsequent two sections respectively present Neurokernel’s programming model and detail its API. To illustrate the use of Neurokernel’s API, we then describe its use to integrate independently developed models of the retina and lamina neuropils in the fly’s visual system. We also assess Neurokernel’s ability to exploit technology for accelerated data transmission between multiple GPUs in benchmarks of its module communication services. Finally, we compare Neurokernel to other computational projects directed at reverse engineering the function of neural circuits and discuss the project’s long-term goals.

## Framework Design and Features

### Modeling the Fruit Fly Brain

**Fig 1:**
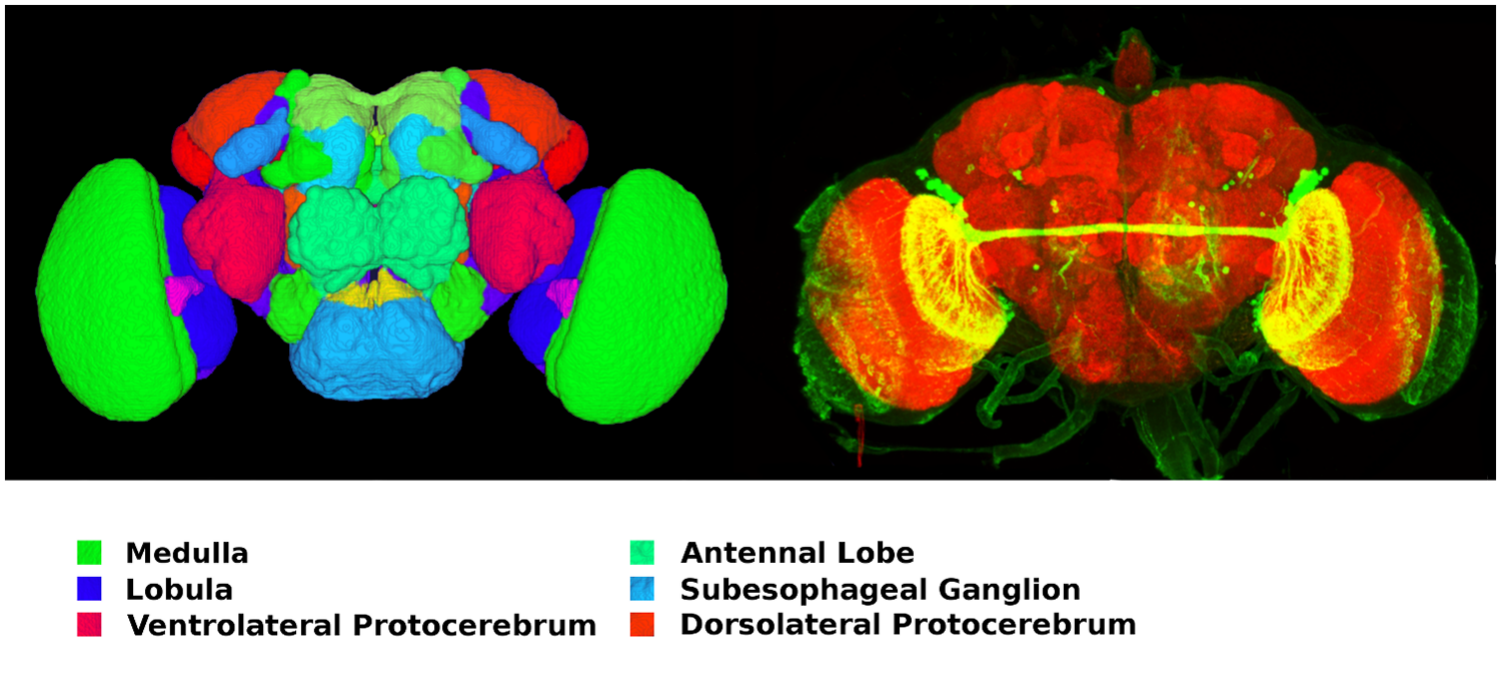
Modular structure of fruit fly brain. Individual neuropils are identified by different colors in the left-hand figure, with the names of several major neuropils listed. Most neuropils are paired across the fly’s two hemispheres. The right-hand figure depicts a tract of neuronal axons connecting neuropils across hemispheres highlighted in yellow (image created using data and software from [26–28], reproduced with permission).

Analysis of the *Drosophila* connectome has revealed that its brain can be decomposed into fewer than 50 distinct neural circuits, most of which correspond to anatomically distinct regions in the fly brain [4]. These regions, or neuropils, include sensory circuits such as the olfactory system’s antennal lobe and the visual system’s lamina and medulla, as well as control and integration neuropils such as the protocerebral bridge and ellipsoid body (Fig 1). Neuropils range in size from about 6,000 neurons (lamina) to 40,000 neurons (medulla). Most of these modules are referred to as local processing units (LPUs) because they are characterized by unique populations of local neurons whose processes are restricted to specific neuropils.

The axons of an LPU’s local neurons and the synaptic connections between them and other neurons in the LPU constitute an internal pattern of connectivity that is distinct from the bundles, or tracts, of projection neuron processes that transmit data to neurons in other LPUs (Fig 1); this suggests that an LPU’s local neuron population and synaptic connections largely determine its functional properties. While the connection densities within and between LPUs is not fully known, the total strength of connections between LPUs (defined in terms of total numbers of dendritic and axonal terminals for all projection neurons linking a LPU with other LPUs) has been observed to vary between 600 and 44,000 for a sample of 13,000 projection neurons in the adult *Drosophila* brain [25]. The fruit fly brain also comprises modules referred to as hubs that contain no local neurons; they appear to serve as communication relays between different LPUs.

In contrast to a purely anatomical subdivision, the decomposition of the brain into functional modules casts the problem of reverse engineering the brain as one of discovering the information processing performed by each individual LPU and determining how specific patterns of axonal connectivity between these LPUs integrates them into functional subsystems. Modeling both these functional modules and the connectivity patterns that link them independent of the internal design of each module is a fundamental requirement of Neurokernel’s architecture.

### Architecture of the Neurokernel

We refer to our software framework for fruit fly brain emulation as a *kernel* because it aims to provide two classes of functions associated with traditional computer operating systems [29]: it must serve as a *resource allocator* that enables the scalable use of parallel computing resources to accelerate the execution of an emulation, and it must serve as an *extended machine* that provides software services and interfaces that can be programmed to emulate and integrate functional modules in the fly brain.

**Fig 2:**
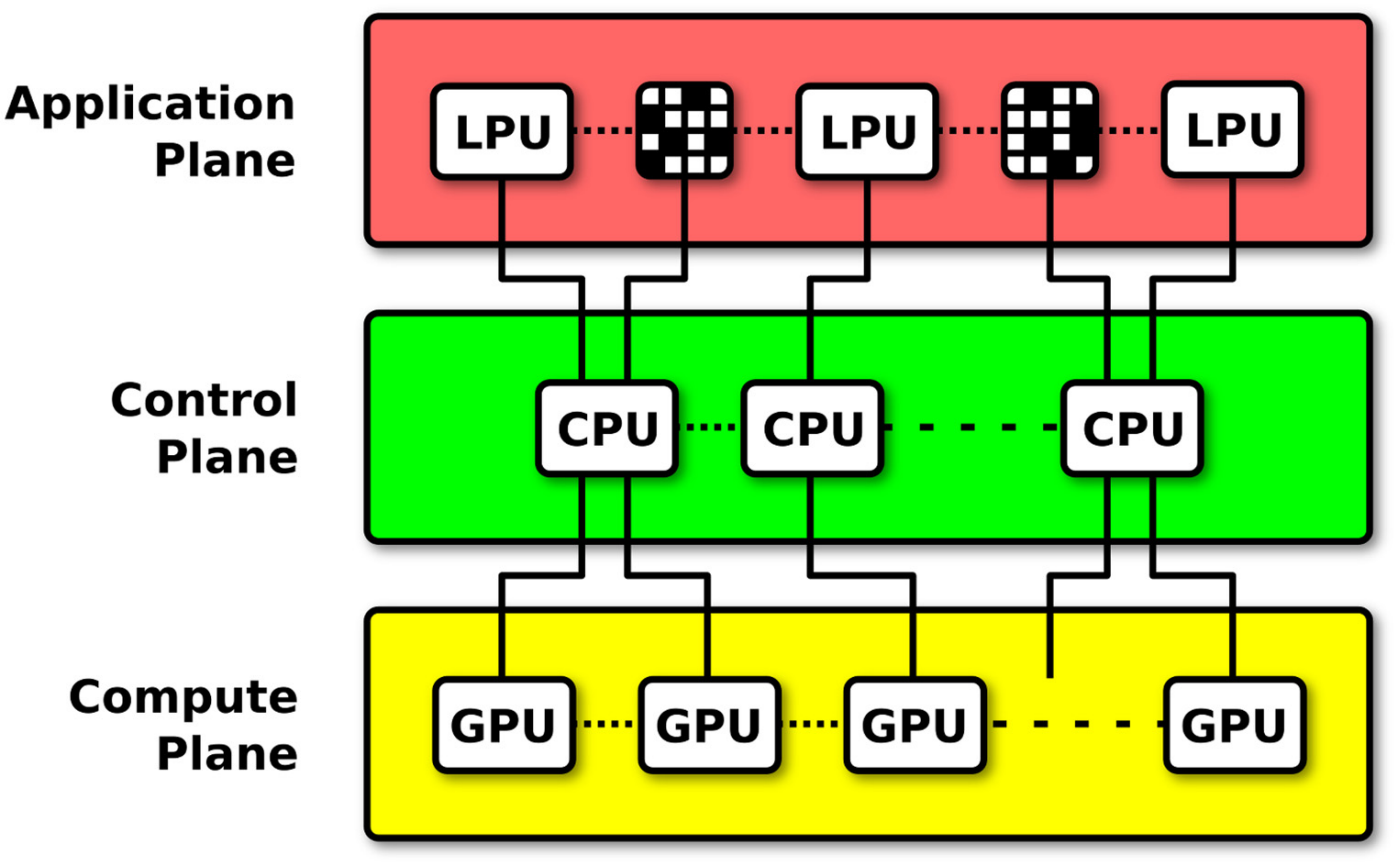
The three-plane structure of the Neurokernel architecture is based on the principle of separation of time scales. The application plane provides support for hardware-independent specification of LPUs and their interconnects. Services that implement the neural primitives and computing methods required to execute neural circuit model instantiations on GPUs are provided by the compute plane. Translation or mapping of specified model components to the methods provided by the compute plane and management of multiple GPUs and communication resources is performed by the control plane operating on a cluster of CPUs.

Neurokernel’s architectural design consists of three planes that separate between the time scales of a model’s representation and its execution on multiple parallel processors (Fig 2). Each plane exposes a vertical API that provides abstractions/services of that plane to higher level planes; this enables development of new features within one plane while minimizing the need to modify code associated with other planes. Services that implement the computational primitives and numerical methods required to execute supported models on parallel processors are provided by the framework’s *compute plane*. Translation or mapping of a models’ specified components to the methods provided by the compute plane and management of the parallel hardware and data communication resources required to efficiently execute a model is performed by Neurokernel’s *control plane*. Finally, the framework’s *application plane* provides support for specification of neural circuit models, connectivity patterns, and interfaces that enable independently developed models of the fly brain’s functional subsystems to be interconnected; we describe these interfaces in greater detail in the Application Programming Interface section.

### Neurokernel Programming Model

#### Interface Configuration

**Fig 3:**
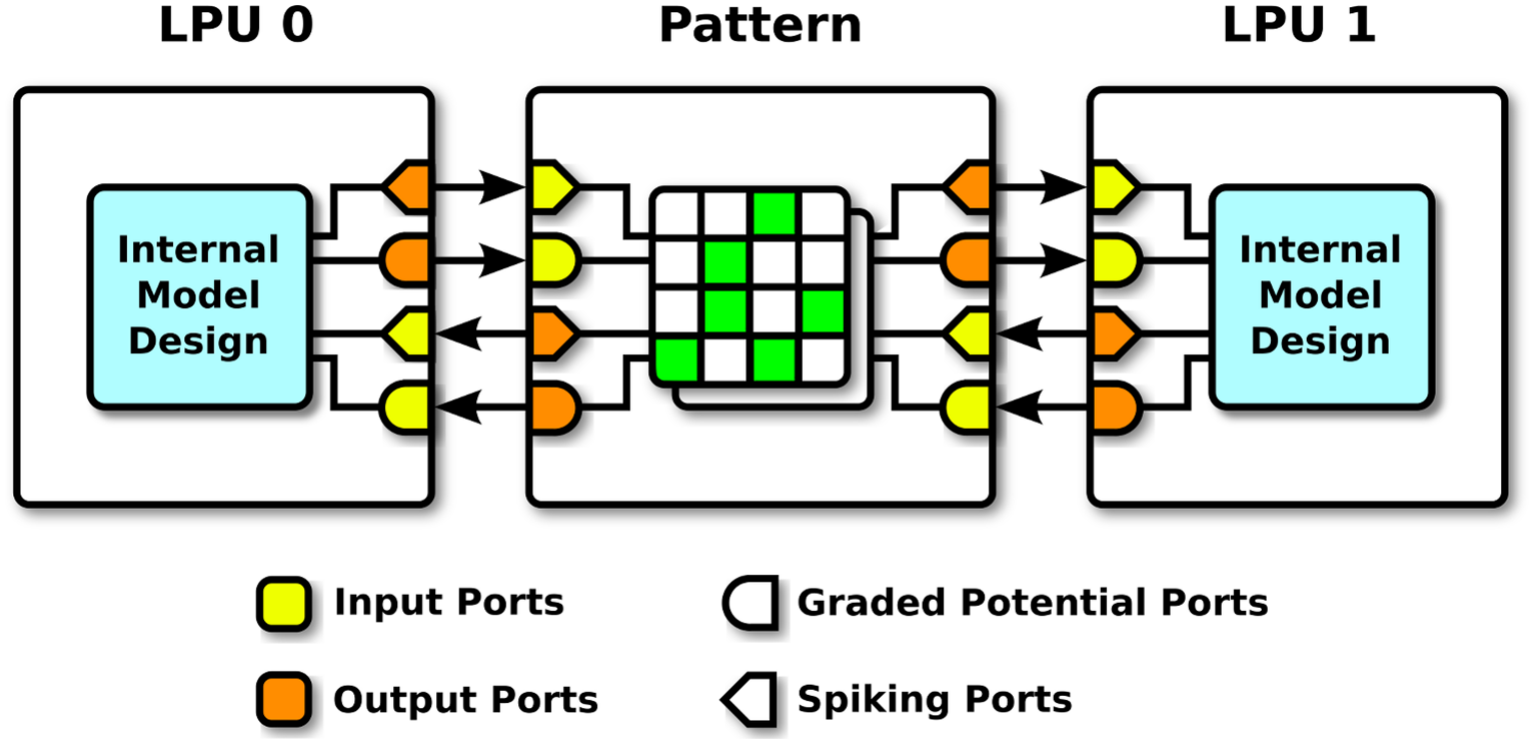
Neurokernel programming model. An LPU model’s internal components (cyan) are exposed via input and output ports (yellow and orange). Connections between LPUs are described by patterns (green) that link the ports of one LPU to those of another. Connections may only be defined between ports of the same transmission type.

**Fig 4:**
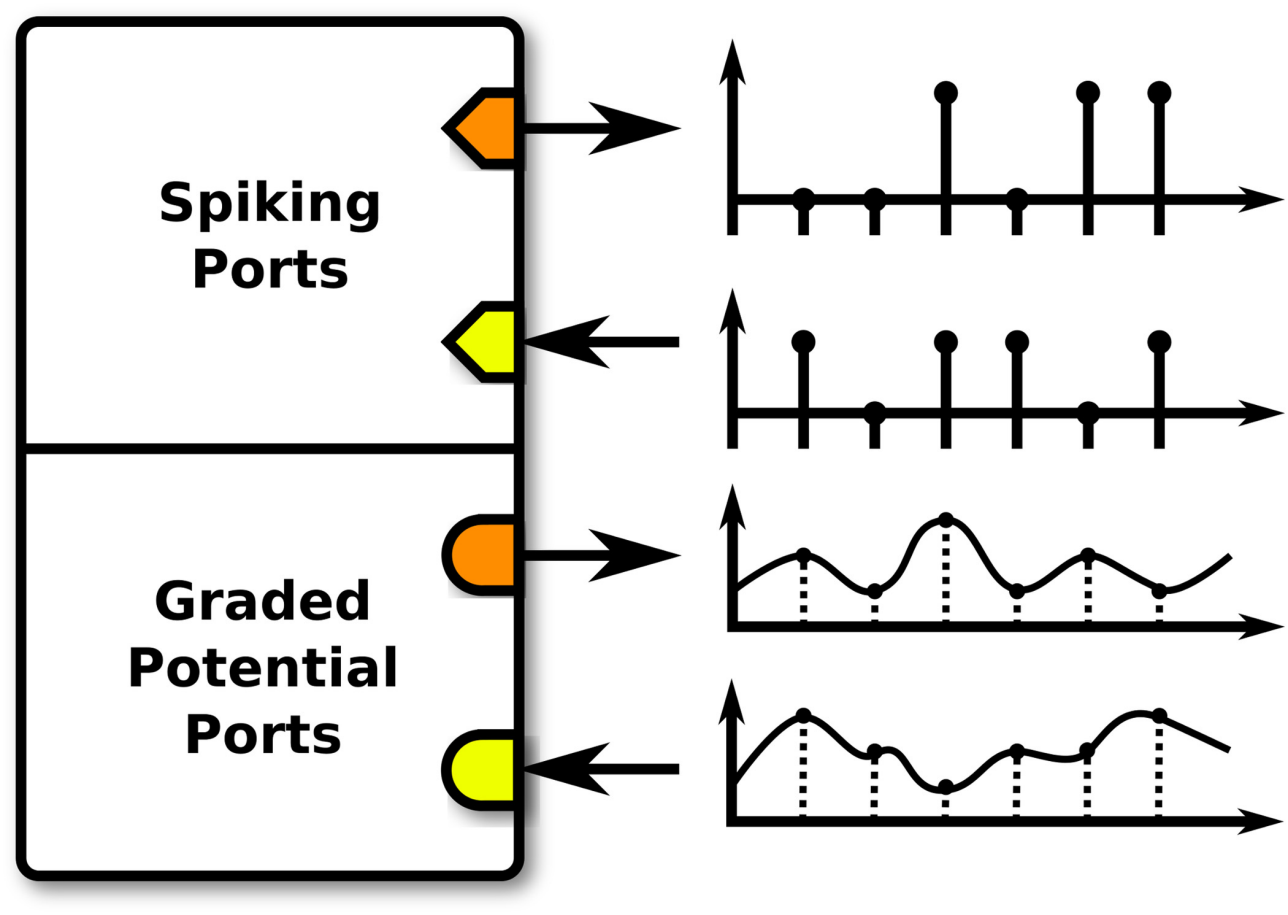
LPU interface. Each communication port must either receive input (yellow) or emit output (orange), and must either transmit spikes (diamonds) or graded potentials (circles).

A key aspect of Neurokernel’s design is the separation it imposes between the internal processing performed by an LPU model and how that model communicates with other models (Fig 3). Neurokernel’s programming model requires that one specifies how an LPU’s interface is configured and connected to those of other LPUs. The interface of an LPU must be described exclusively in terms of communication ports that either transmit data to or receive data from ports exposed by other LPUs after each execution step. Each port must be configured either to receive input or emit output, and must be configured to either accept spike data represented as boolean values or graded potential data represented as floating point values (Fig 4). Both of these settings are mutually exclusive; a single port may not both receive input and emit output, nor may it accept both spike and graded potential data. Ports may be connected to arbitrary internal components of an LPU; a graded potential port, for example, need not be associated with a neuron model’s membrane voltage. Ports are uniquely specified relative to other ports within an interface using a path-like identifier syntax to facilitate hierarchical organization of large numbers of ports (Table 1).

**Table 1:** Path-like port identifier and selector syntax examples. In these examples, the identifier level strings med and L1 are chosen to respectively denote an LPU and a neuron within that LPU. An interface designer may select whichever level strings are deemed suitable to label ports in an interface, however.

Identifier/Selector	Comments
/med/L1[0]	selects a single port
/med/L1/0	equivalent to /med/L1[0]
/med+/L1[0]	equivalent to /med/L1[0]
/med/[L1,L2][0]	selects two ports
/med/L1[0, 1]	another example of two ports
/med/L1[0],/med/L1[1]	equivalent to /med/L1[0, 1]
/med/L1[0:10]	selects ten ports
/med/L1/*	selects all ports starting with /med/L1
(/med/L1,/med/L2)+[0]	equivalent to /med/[L1,L2][0]
/med/[L1,L2].+[0:2]	equivalent to /med/L1[0],/med/L2[1]

#### Pattern Configuration

A single LPU may potentially be connected to many other LPUs; these connections must be expressed as patterns between pairs of LPUs (Fig 3). Each pattern must be expressed in terms of (1) two interfaces—each comprising a set of ports—between which connections may be defined, (2) the actual connections between individual ports in the two interfaces (Table 2), and (3) the attributes of each port in the pattern’s interfaces (Table 3).

**Table 2:** Example of connections between ports in two LPUs respectively denoted lam and med. An instance of the Pattern class comprises these connections and the port attributes in Table 3.

Source Port	Destination Port
/lam[0]	/med[0]
/lam[0]	/med[1]
/lam[1]	/med[2]
/med[3]	/lam[3]
/med[4]	/lam[4]
/med[4]	/lam[5]

**Table 3:** Attributes of the ports in the connectivity pattern described in Table 2.

Port	Interface	I/O	Port Type
/lam[0]	0	in	graded potential
/lam[1]	0	in	graded potential
/lam[2]	0	out	graded potential
/lam[3]	0	out	spiking
/lam[4]	0	out	spiking
/lam[5]	0	out	spiking
/med[0]	1	out	graded potential
/med[1]	1	out	graded potential
/med[2]	1	out	graded potential
/med[3]	1	in	spiking
/med[4]	1	in	spiking

Port attributes are used by Neurokernel to determine compatibility between LPU and pattern objects. To provide LPU designers with the freedom to determine how to multiplex input data from multiple sources within an LPU, Neurokernel does not permit multiple input ports in a pattern to be connected to a single output port. Input ports in a pattern may be connected to multiple output ports. It should be noted that the connections defined by an inter-LPU connectivity pattern do not represent synaptic models; any synapses comprised by a brain model must be a part of the design of a constituent LPU and connected to the LPU’s ports in order to either receive or transmit data from or to modeling components in other LPUs.

### Application Programming Interface

In contrast to other currently available GPU-based neural emulation packages [30–33], Neurokernel is implemented entirely in Python, a high-level language with a rich ecosystem of scientific packages that has enjoyed increasing popularity in neuroscience research. Although GPUs can be directly programmed using frameworks such as NVIDIA CUDA and OpenCL, the difficulty of writing and optimizing code using these frameworks exclusively has led to the development of packages that enable run-time code generation (RTCG) using higher level languages [34]. Neurokernel uses the PyCUDA package to provide RTCG support for NVIDIA’s GPU hardware without forgoing the development advantages afforded by Python [35].

To make use of Neurokernel’s LPU API, all LPU models must subclass a base Python class called Module that provides LPU designers with the freedom to organize the internal structure of their model implementations as they see fit independent of the LPU interface configuration. Implementation of a Neurokernel-compatible LPU requires that (1) the LPU be uniquely identified relative to all other LPUs to which it may be connected in a subsystem or whole-brain emulation, (2) the execution of all operations comprised by a single step of the LPU’s emulation be performed by invocation of a single method called run_step(), and that (3) the LPU’s interface be configured as described in the Interface Configuration subsection.

An instantiated LPU’s graded potential and spiking ports are respectively associated with GPU data arrays that Neurokernel accesses to transmit data between LPUs during emulation execution; LPU designers are responsible for reading the data elements associated with input ports and populating the elements associated with output ports in the run_step() method. Modeling components that do not communicate with other LPUs and the internal connectivity patterns defined between them are not made accessible through the LPU’s interface (Fig 3).

Inter-LPU connectivity patterns correspond to the connections described by the tracts depicted in Fig 1. These are represented by a tensor-like class called Pattern that contains the port and connection data described in the Pattern Configuration subsection. To conserve memory, only existing connections are stored in a Pattern instance. In addition to manually constructing inter-LPU connectivity patterns using the configuration methods provided by the Pattern class, Neurokernel also supports loading connectivity patterns from CSV, GEXF, or XML files using a schema similar to NeuroML [36] with components that enable the specification of LPUs, connectivity patterns, and the ports they expose. Inter-LPU connections currently remain static throughout an emulation; future versions of Neurokernel will support dynamic instantiation and removal of connections while a model is being executed.

The designer of an LPU is responsible for associating ports with internal components that either consume input data or emit output data. Neurokernel provides a class called GPUPortMapper that maps port identifiers to GPU data arrays; by default, each Module instance contains two GPUPortMapper instances that respectively associate the LPU’s ports with arrays containing graded potential and spike values. After each invocation of the LPU’s run_step() method, data within these arrays associated with the LPU’s output ports is automatically transmitted to the port data arrays of destination LPUs, while input data from source LPUs is automatically inserted into those elements associated with the LPU’s input ports (Table 4).

**Table 4:** Example of input and output data mapped to and from data arrays by the GPUPortMapper class for the ports comprised by interface 0 in the pattern described in Tables 2 and 3.

Graded Potential Ports			Spiking Ports		
Port	Array Index	Array Data	Port	Array Index	Array Data
/lam[0]	0	0.71	/lam[3]	0	1
/lam[1]	1	0.83	/lam[4]	1	0
/lam[2]	2	0.52	/lam[5]	2	1

In addition to the classes that represent LPUs and inter-LPU connectivity patterns, Neurokernel provides an emulation manager class called Manager that provides services for configuring LPU classes, connecting them with specified connectivity patterns, and determining how to route data between LPUs based upon those patterns. The manager class hides the process and communication management performed by OpenMPI so as to obviate the need for model designers to directly interact with the traditional MPI job launching interface. Once an emulation has been fully configured via the manager class, it may be executed for a specified interval of time or for a specified number of steps.

**Fig 5:**
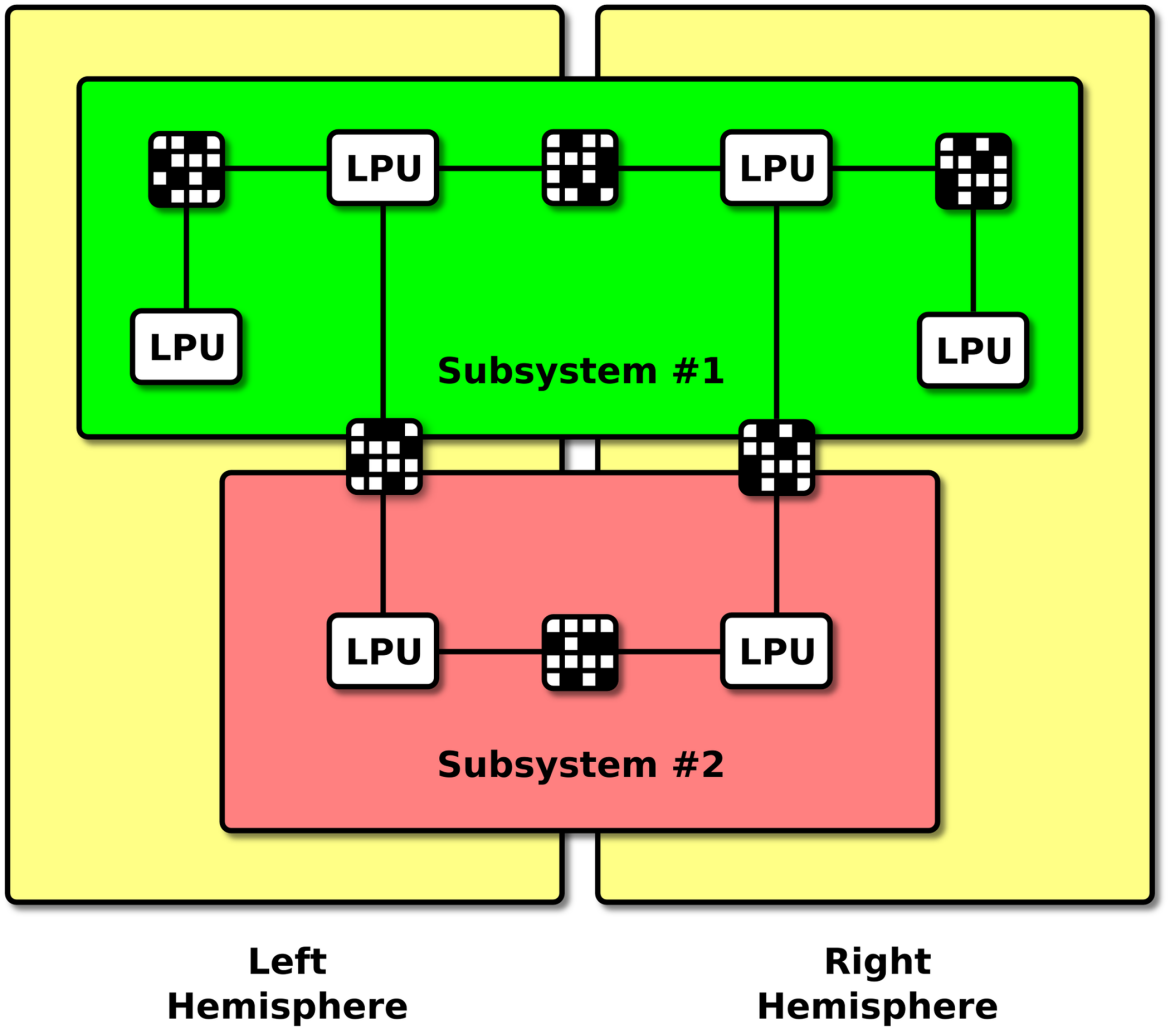
Neurokernel brain modeling architectural hierarchy. Independently developed LPUs and connectivity patterns may be composed into subsystems (red, green) which may in turn be connected to other subsystems to construct a model of the whole brain (yellow).

Apart from the API requirements discussed above, Neurokernel currently places no explicit restrictions upon an LPU model’s internal implementation, how it interacts with available GPUs, how LPUs record their output, or the topology of interconnections between different LPUs; compatible LPUs and inter-LPU patterns may be arbitrarily composed to construct subsystems (Fig 5). It should be noted that the current LPU interface is not intended to be final; we anticipate its gradual extension to support communication between models that more accurately account for the range of interactions that occur within the fruit fly’s brain.

Neurokernel’s compute plane currently provides GPU-based implementations for several common neuron and synapse models. Supported neuron models include the Leaky Integrate-and-Fire, Hodgkin-Huxley, and Morris-Lecar point neuron models, as well as a stochastic model of the photoreceptors in the fly retina. Alpha function and conductance-based synaptic models are also supported. These modeling components may be used to construct and execute LPUs without writing any Python code by specifying an LPU’s design declaratively as a graph stored in GEXF, an XML format for storing a property graph supported by various graph processing libraries. Neurokernel does not restrict LPU model developers to using the above models; additional modeling components may be added to the compute plane as plugins.

Communication between LPU instances in a running Neurokernel emulation is performed using MPI to enable brain emulations to take advantage of multiple GPUs hosted either on single computer or a computer cluster. Neurokernel uses OpenMPI [37] to provide accelerated access between GPUs that support NVIDIA’s GPUDirect Peer-to-Peer technology [38, 39] when the source and destination memory locations of an MPI data transfer are both in GPU memory. Neurokernel-based models are executed in a bulk synchronous fashion; each LPU’s execution step is executed asynchronously relative to other LPUs’ execution steps, but data associated with the output ports of all connected LPUs must be propagated to their respective destinations before those LPUs can proceed to the next execution step. Since data is transmitted between connected LPUs at every execution step, the output ports of all LPUs are effectively sampled at the same rate. Individual LPUs may perform internal computations at a finer time resolution, provided that they update their output port data arrays at the end of each invocation of their run_step() methods.

### Using the Neurokernel API

This section illustrates how to use the Neurokernel classes described in the Application Programming Interface section to construct and execute an emulation consisting of multiple connected LPUs. The section assumes that Neurokernel and its relevant dependencies (including OpenMPI) have already been installed on a system containing multiple GPUs. First, we import several required Python modules; the mpi_relaunch module provided by Neurokernel sets up the MPI environment required to enable communication between LPUs.

import neurokernel.mpi_relaunch
from mpi4py import MPI
import numpy as np
import pycuda.gpuarray as gpuarray
from neurokernel.mpi import setup_logger
from neurokernel.core_gpu import Module, Manager
from neurokernel.pattern import Pattern
from neurokernel.plsel import Selector, SelectorMethods

Next, we create a subclass of Module whose run_step() method accesses the class instance’s port data arrays; the example below generates random graded potential and spiking output port data.

class MyModule (Module):
  ″ ″ ″
  *Example of derived module class*.
  ″ ″ ″
  def run_step (self):
    *# Call the run_step() method of the parent class (Module)*:
    super (MyModule, self).run_step()
    *# Log input graded potential data*:
    self.log_info(‘input gpot port data: ‘+\ str(self.pm[‘gpot’][self.in_gpot_ports]))
    *# Log input spike data*:
    self.log_info (‘input spike port data: ‘+\ str (self.pm[‘spike’][self.in_spike_ports]))
    *# Output random graded potential data*:
    out_gpot_data = \ gpuarray.to_gpu(np.random.rand(len(self.out_gpot_ports)))
    self.pm[‘gpot’][self.out_gpot_ports] = out_gpot_data
    self.log_info (‘output gpot port data: ‘+str (out_gpot_data))
    *# Output spikes to randomly selected output ports*:
    out_spike_data = \ gpuarray.to_gpu(np.random.randint(0, 2, len (self.out_spike_ports)))
    self.pm[‘spike’][self.out_spike_ports] = out_spike_data
    self.log_info(‘output spike port data: ‘+str(out_spike_data))

The data arrays associated with an LPU’s ports may be accessed using their path-like identifiers via two instances of the GPUPortMapper class stored in the self.pm attribute. Updated data associated with output ports is propagated to the relevant destination LPUs by Neurokernel before the next iteration of the emulation’s execution.

To connect two LPUs, we specify the ports to be exposed by each LPU using path-like selectors. The example below describes the interfaces for two LPUs that each expose two graded potential input ports, two graded potential output ports, two spiking input ports, and two spiking output ports. Selector is a convenience class that provides methods and overloaded operators for combining and manipulating sets of validated port identifiers. For example, Selector(‘/a/in/gpot[0:2]’) corresponds to the set of two input graded potential port identifiers /a/in/gpot[0] and /a/in/gpot[1]. Additional methods for manipulating port identifiers are provided by the SelectorMethods class.

*# Define input graded potential, output graded potential*,
*# input spiking, and output spiking ports for LPUS ‘a’ and ‘b’*:
m1_sel_in_gpot = Selector(‘/a/in/gpot[0:2]’)
m1_sel_out_gpot = Selector(‘/a/out/gpot[0:2]’)
m1_sel_in_spike = Selector(‘/a/in/spike[0:2]’)
m1_sel_out_spike = Selector(‘/a/out/spike[0:2]’)
m2_sel_in_gpot = Selector(‘/b/in/gpot[0:2]’)
m2_sel_out_gpot = Selector(‘/b/out/gpot[0:2]’)
m2_sel_in_spike = Selector(‘/b/in/spike[0:2]’)
m2_sel_out_spike = Selector(‘/b/out/spike[0:2]’)
*# Combine selectors to obtain sets of all input, output*,
*# graded potential, and spiking ports for the two LPUs*:
m1_sel = m1_sel_in_gpot+m1_sel_out_gpot+\ m1_sel_in_spike+m1_sel_out_spike
m1_sel_in = m1_sel_in_gpot+m1_sel_in_spike
m1_sel_out = m1_sel_out_gpot+m1_sel_out_spike
m1_sel_gpot = m1_sel_in_gpot+m1_sel_out_gpot
m1_sel_spike = m1_sel_in_spike+m1_sel_out_spike
m2_sel = m2_sel_in_gpot+m2_sel_out_gpot +\ m2_sel_in_spike+m2_sel_out_spike
m2_sel_in = m2_sel_in_gpot+m2_sel_in_spike
m2_sel_out = m2_sel_out_gpot+m2_sel_out_spike
m2_sel_gpot = m2_sel_in_gpot+m2_sel_out_gpot
m2_sel_spike = m2_sel_in_spike+m2_sel_out_spike
*# Count the number of graded potential and*
*# spiking ports exposed by each LPU*:
N1_gpot = SelectorMethods.count_ports(m1_sel_gpot)
N1_spike = SelectorMethods.count_ports(m1_sel_spike)
N2_gpot = SelectorMethods.count_ports(m2_sel_gpot)
N2_spike = SelectorMethods.count_ports(m2_sel_spike)

Using the above LPU interface data, we construct an inter-LPU connectivity pattern by instantiating the Pattern class, setting its port input/output and transmission types, and populating it with connections:

*# Initialize connectivity pattern that can link*
*# ports in m1_sel with ports in m2_sel*:
pat12 = Pattern(m1_sel, m2_sel)
*# Set the input/output and transmission type attributes of each port in the pattern’s two interfaces*:
pat12.interface[m1_sel_out_gpot] = [0, ‘in’, ‘gpot’]
pat12.interface[m1_sel_in_gpot] = [0, ‘out’, ‘gpot’]
pat12.interface[m1_sel_out_spike] = [0, ‘in’, ‘spike’]
pat12.interface[m1_sel_in_spike] = [0, ‘out’, ‘spike’]
pat12.interface[m2_sel_in_gpot] = [1, ‘out’, ‘gpot’]
pat12.interface[m2_sel_out_gpot] = [1, ‘in’, ‘gpot’]
pat12.interface[m2_sel_in_spike] = [1, ‘out’, ‘spike’]
pat12.interface[m2_sel_out_spike] = [1, ‘in’, ‘spike’]
*# Create the connections between ports*:
pat12[‘/a/out/gpot[0]’, ‘/b/in/gpot[0]’] = 1
pat12[‘/a/out/gpot[1]’, ‘/b/in/gpot[1]’] = 1
pat12[‘/b/out/gpot[0]’, ‘/a/in/gpot[0]’] = 1
pat12[‘/b/out/gpot[1]’, ‘/a/in/gpot[1]’] = 1
pat12[‘/a/out/spike[0]’, ‘/b/in/spike[0]’] = 1
pat12[‘/a/out/spike[1]’, ‘/b/in/spike[1]’] = 1
pat12[‘/b/out/spike[0]’, ‘/a/in/spike[0]’] = 1
pat12[‘/b/out/spike[1]’, ‘/a/in/spike[1]’] = 1

We can then pass the defined LPU class and the parameters to be used during instantiation to a Manager class instance that connects them together with the above pattern. The setup_logger function may be used to enable output of log messages generated during execution:

logger = setup_logger(screen = True, file_name=‘neurokernel.log’, mpi_comm = MPI.COMM_WORLD, multiline = True)
man = Manager()
m1_id = ‘m1’
man.add(MyModule, m1_id, m1_sel, m1_sel in, m1_sel_out, m1_sel_gpot, m1_sel_spike, np.zeros(N1_gpot, dtype = np.double), np.zeros(N1_spike, dtype = int), device = 0)
m2_id = ‘m2’
man.add(MyModule, m2_id, m2_sel, m2_sel_in, m2_sel_out, m2_sel_gpot, m2_sel_spike, np.zeros(N2_gpot, dtype = np.double), np.zeros(N2_spike, dtype = int), device = 1)
man.connect(m1_id, m2_id, pat12, 0, 1)

After all LPUs and connectivity patterns are provided to the manager, the emulation may be executed for a specified number of steps as follows. Neurokernel uses the dynamic process creation feature of MPI-2 supported by OpenMPI to automatically spawn as many MPI processes are needed to run the emulation:

*# Compute number of execution steps given emulation duration*
*# and time step (both in seconds)*:
duration = 10.0
dt = 1e-2
steps = int(duration/dt)
man.spawn()
man.start(steps)
man.wait()

## Results

To evaluate Neurokernel’s ability to facilitate interfacing of functional brain modules that can be executed on GPUs, we employed Neurokernel’s programming model to interconnect independently developed LPUs in the fruit fly’s early visual system to provide insights into the representation and processing of the visual field by the cascaded LPUs. We also evaluated Neurokernel’s scaling of communication performance in simple configurations of the architecture parameterized by numbers of ports and LPUs.

The scope of the effort to reverse engineer the fly brain and the need to support the revision of brain models in light of new data requires a structured means of advancing and documenting the evolution of those models and the framework required to support them. To this end, the Neurokernel project employs Requests for Comments documents (RFCs) as a tool for advancing the designs of both Neurokernel’s architecture and the LPU models built to use it. IPython notebooks and RFCs [40, 41] containing detailed descriptions of the models of the visual system LPUs described below and their execution performance on multiple GPUs are publicly available on the project website http://neurokernel.github.io/docs.html.

### Integration of Independently Developed LPU Models

The integrated early visual system model we considered consists of models of the fruit fly’s retina and lamina. The retina model comprises a hexagonal array of 721 ommatidia, each of which contains 6 photoreceptor neurons. The photoreceptor model employs a stochastic model of how light input (photons) produce a membrane potential output. Each photoreceptor consists of 30,000 microvilli modeled by 15 equations per microvillus, a photon absorption model, and a model of how the aggregate microvilli contributions produce the photoreceptor’s membrane potential [41]; the entire retina model employs a total of about 1.95 billion equations. The lamina model consists of 4,326 Morris-Lecar neurons configured to not emit action potentials and about 50,000 conductance-based inhibitory synapses expressing histamine [40]. The LPUs were linked by 4,326 feed-forward connections from the retina to the lamina; the connections from the retina to the lamina were configured to map output ports exposed by the retina to input ports in the lamina based upon the neural superposition rule [42]. The source code for the visual system model is available at http://github.com/neurokernel/retina-lamina

**Fig 6:**
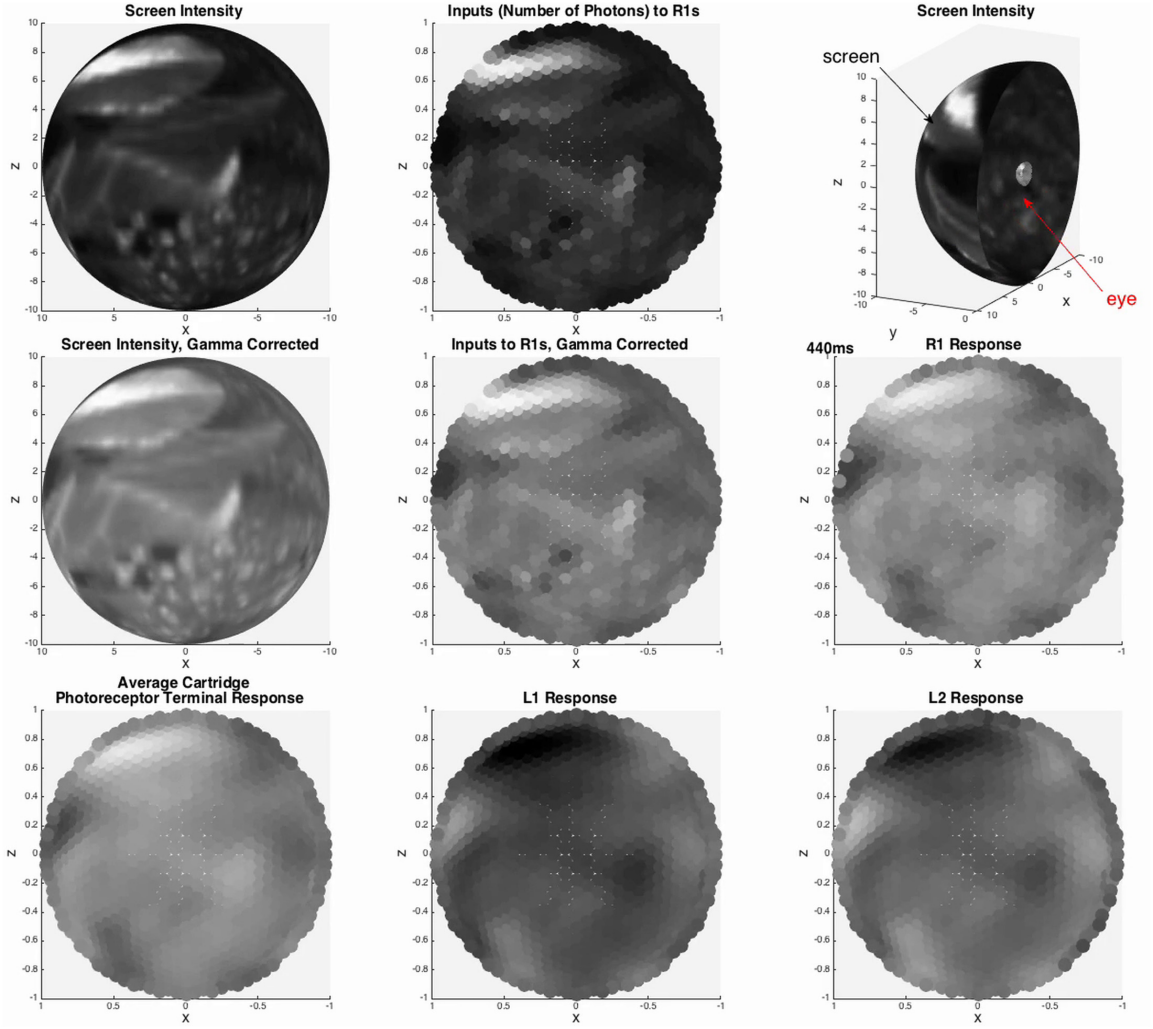
Example of natural input to the combined retina/lamina model. The hexagonal tiling depicts the array of ommatidia in the retina and the corresponding retinotopic cartridges in the lamina. Outputs of select photoreceptors in the retina (R1) that are fed to neurons in the lamina and outputs of specific neurons in the lamina (L1, L2) are also depicted.

The combined retina and lamina models were executed on up to 4 Tesla K20Xm NVIDIA GPUs with an 8 second natural video scene provided as input to the retinal model’s photoreceptors. The computed membrane potentials of specific photoreceptors in each retinal ommatidium and of select neurons in each cartridge of the lamina were recorded (Fig 6); videos of the computed potentials are included in the supporting information (S1 Video). In this example, the observed R1 photoreceptor outputs demonstrate the preservation of visual information received from the retina by the lamina LPU. The L1 and L2 lamina neuron outputs demonstrate the signal inversion taking place in the two pathways shaping the motion detection circuitry of the fly. These initial results illustrate how Neurokernel’s API enables LPU model designers to treat their models as neurocomputing modules that may be combined into complex information processing pipelines whose input/output properties may be obtained and evaluated.

### Module Communication Performance

We compared the performance of emulations in which port data stored in GPU memory is copied to and from host memory for traditional network-based transmission by OpenMPI to that of emulations in which port data stored in GPU memory is directly passed to OpenMPI’s communication functions. The latter functions enabled OpenMPI to use NVIDIA’s GPUDirect Peer-to-Peer technology to perform accelerated transmission of data between GPUs whose hardware supports the technology by bypassing the host system’s CPU and memory [39]. All tests discussed below were performed on a host containing 2 Intel Xeon 6-core E5-2620 CPUs, 32 Gb of RAM, and 4 NVIDIA Tesla K20Xm GPUs running Ubuntu Linux 14.04, NVIDIA CUDA 7.0, and OpenMPI 1.8.5 built with CUDA support.

#### Scaling over Number of LPU Output Ports

To evaluate how well inter-LPU communication scales over the number of ports exposed by an LPU on a multi-GPU machine, we constructed and ran emulations comprising multiple connected instances of an LPU class with an empty run_step() method (see the Application Programming Interface section) and measured (1) the average time taken per execution step to synchronize the data exposed by the output ports in each of two connected LPUs with their respective destination input ports; (2) the average throughput per execution step (in terms of number of port data elements transmitted per second) of the synchronization, where each port is stored either as a 32-bit integer or double-precision floating point number (both of which occupy 8 bytes).

We initially examined how the above performance metrics scaled over the number of output ports exposed by each LPU in a 2-LPU emulation and over the number of LPUs in an emulation where each LPU is connected to every other LPU and the total number of output ports exposed by each LPU is fixed. We compared the performance for scenarios where data in GPU memory is directly exposed to OpenMPI to that for scenarios where the data is copied to the host memory prior to transmission; the former scenarios enabled OpenMPI to accelerate data transmission between GPUs using NVIDIA’s GPUDirect Peer-to-Peer technology. The metrics for each set of parameters were averaged over 3 trials; the emulation was executed for 500 steps during each trial.

**Fig 7:**
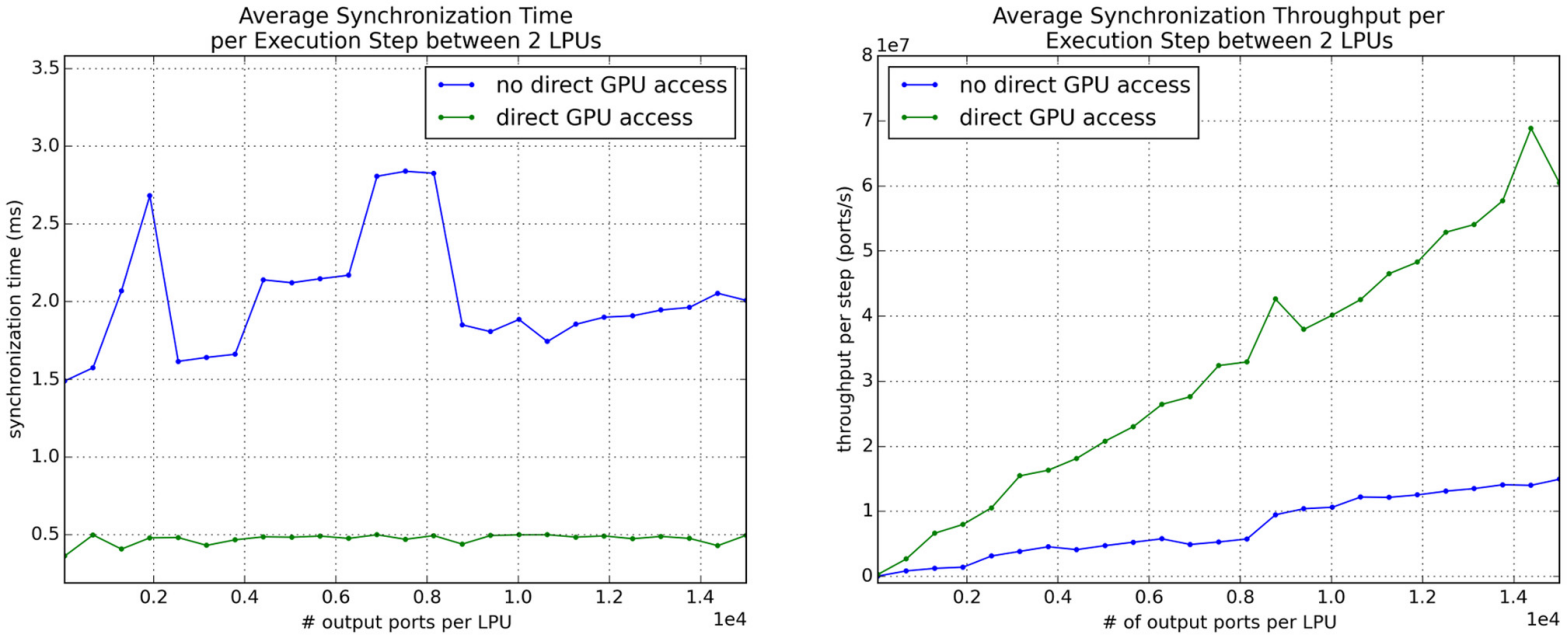
Synchronization performance for an emulation comprising 2 interconnected LPUs accessing 2 different GPUs on the same host scaled over number of output ports exposed by each LPU. The number of output ports was varied over 25 equally spaced values between 50 and 15,000. The plot on the left depicts average synchronization time per execution step, while the plot on the right depicts average synchronization throughput (in number of ports per unit time) per execution step.

**Fig 8:**
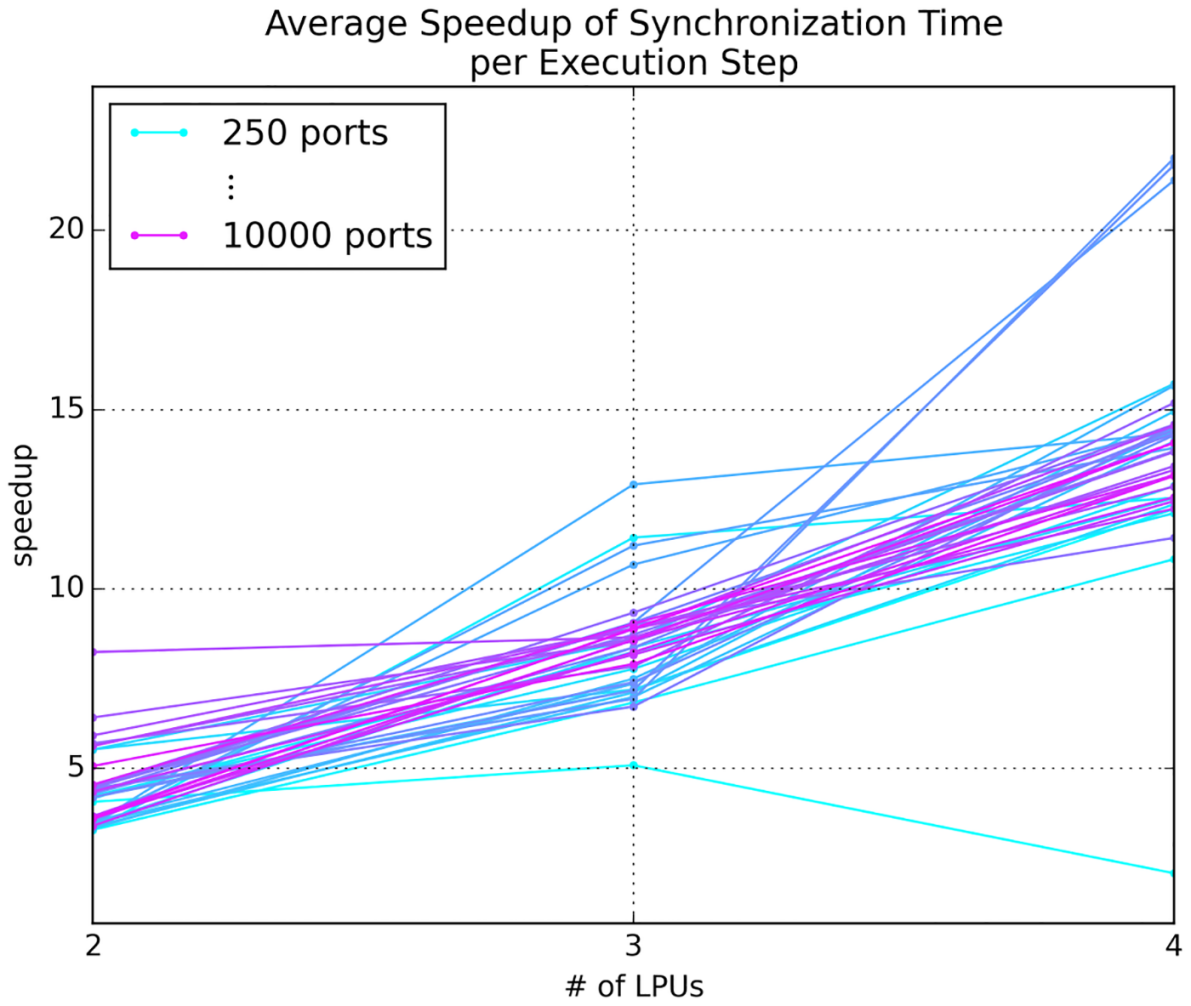
Speedup of average synchronization time per execution step for an emulation scaled over number of LPUs, where each LPU is mapped to a single GPU. The total number of output ports exposed by each LPU was varied between 250 and 10,000 at 250 port intervals.

The scaling of performance over number of ports depicted in Fig 7 clearly illustrate the ability of GPU-to-GPU communication between locally hosted GPUs to ensure that increasing the number of ports exposed by an LPU does not increase model execution time for numbers of ports similar to the numbers of neurons in actual LPUs. We also observed noticeable speedups in synchronization time for scenarios using more than 2 GPUs as the number of ports exposed by each LPU is increased (Fig 8). As the number of GPUs in use reached the maximum available in our test system, overall speedup diminished; this appears to be due to gradual saturation of the host’s PCI bus.

#### Scaling over Number of LPUs

**Fig 9:**
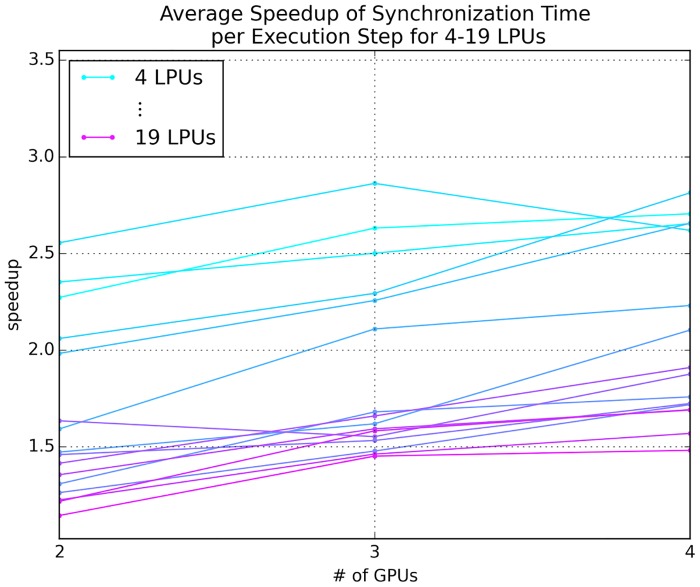
Synchronization performance for an emulation comprising between 4 and 19 interconnected LPUs selected from the central complex, olfactory, and vision systems partitioned over 2 to 4 GPUs on the same host.

Current research on the fruit fly brain is mainly focused on LPUs in the fly’s central complex and olfactory and vision systems. Since the interplay between these systems will be key to increasing understanding of multisensory integration and how sensory data might inform behavior mediated by the central complex, we examined how well Neurokernel’s communication mechanism performs in scenarios where LPUs from these three systems are successively added to a multi-LPU emulation. Starting with the pair of LPUs with the largest number of inter-LPU connections, we sorted the 19 LPUs in the above three systems in decreasing order of the number of connections contributed with the addition of each successive LPU and measured the average speedup in synchronization time per execution step due to direct GPU-to-GPU data. The number of connections for each LPU was based upon estimates from a mesoscopic reconstruction of the fruit fly connectome; these numbers appear in Document S2 of the supplement of [25]. The LPU class instances were designed to send and receive data only; no other computation was performed or benchmarked during execution. To amortize inter-LPU transmission costs, the LPUs were partitioned across the available GPUs using the METIS graph partitioning package [43] to minimize the total edge cut. The speedup afforded by direct GPU-to-GPU data (Fig 9) illustrates that current GPU technology can readily power multi-LPU models based upon currently available connectome data.

## Discussion

In light of their low costs and rapidly increasing power and availability, there is growing interest in leveraging the power of multiple GPUs to support neural simulations with increasingly high computational demands [44–46]. When combined with concomitant increases in fruit fly connectomic knowledge and improvements in electrophysiological techniques, the ongoing advance of GPU technology affords an unprecedented opportunity to emulate an entire brain or nervous system of a computationally tractable organism. The OpenWorm project [47], for instance, is capitalizing on the extremely small number of neurons in the nervous system of the nematode *Caenorhabditis elegans* and the full reconstruction of its connectome [48] to develop an emulation of the entire worm on a computer. A recently started effort is the development of a neuromechanical model called Sibernetic [49] that uses GPUs to power simulation of its body and environment. In a similar vein, Neurokernel stands to enable fly researchers to leverage improving GPU technology to take advantage of the increasing amounts of connectome data produced by ongoing advances in our understanding of the fly brain’s connectivity [4, 16, 17] for designing and testing fly brain models.

Currently available neural simulation software affords researchers with a range of ways of constructing neural circuit models. These include tools that enable models to be explicitly expressed as systems of differential equations [50], structured documents [36], or explicit calls to a high-level programming API [51–53]. They also include tools for defining and manipulating neural connectivity patterns [54–56]. A platform for developing emulations of the entire fruit fly brain, however, must provide programming services for expressing the functional architecture of the whole brain (or its subsystems) in terms of subunits with high-level information processing properties that clearly separate between the internal design of each subunit and how they communicate with each other. Neurokernel’s architecture specifically targets these gaps by providing both the high-level APIs needed to explicitly define and manipulate the architectural elements of brain models as well as the low-level computational substrate required to efficiently execute those models’ implementations on multiple GPUs (see Fig 2).

Existing technologies for interfacing neural models currently provide no native support for the use of GPUs and none of the aforementioned services required to scale over multiple GPU resources. Neurokernel aims to address the problem of model incompatibility in the context of fly brain modeling by ensuring that GPU-based LPU model implementations and inter-LPU connectivity patterns that comply with its APIs are interoperable regardless of their internal implementations.

Despite the impressive performance GPU-based spiking neural network software can currently achieve for simulations comprising increasingly large numbers of neurons and synapses, enabling increasingly detailed fruit fly brain models to efficiently scale over multiple GPUs will require resource allocation and management features that are not yet provided by currently available neural simulation packages. By explicitly providing services and APIs for management of GPU resources, Neurokernel will enable fly brain emulations to benefit from the near-term advantages of scaling over multiple GPUs while leaving the door open to anticipated improvements in GPU technology that can further accelerate the performance of fly brain models.

The challenges of reverse engineering neural systems have spurred a growing number of projects specifically designed to encourage collaborative neuroscience research endeavors. These include technologies for model sharing [36, 57, 58], curation of publicly available electrophysiological data [59], and the construction of comprehensive nervous system models for specific organisms [47]. For collaborative efforts at fruit fly brain modeling to succeed, however, there is a need to both ensure the interoperability of independently developed LPU models without modification of their internal implementations while enforcing a model of the overall brain connectivity architecture. Software packages that enable multiple independently developed neural simulators to execute complex models either by means of communication APIs that simulators must support [60] or through encapsulation of calls to one simulator by a second simulator [61] must be complemented with the flexibility to define and manipulate the emulated connectivity architecture. By imposing mandatory communication interfaces upon models, Neurokernel explicitly ensures that LPU models may be combined with other compatible models to construct subsystem or whole brain emulations.

Neuromorphic platforms whose design is directly inspired by the brain have the potential to execute large-scale neural circuit models at speeds that significantly exceed those achievable with traditional von Neumann computer architectures [62–65]. Increasing support for high-level software interfaces such as PyNN [52] by such platforms raises the possibility of executing highly detailed LPU models on neuromorphic hardware. As neuromorphic technology matures and becomes available to the wider neurocomputing community, we anticipate Neurokernel’s compute plane eventually supporting the use of such hardware alongside and eventually in the place of GPU technology to power whole brain emulations.

Although the Neurokernel project is specifically focused upon reverse engineering the fruit fly brain, the framework’s ability to capitalize upon the structural modularity of the brain and facilitate collaborative modeling stand to benefit efforts to reverse engineer the brains of other model organisms. To this end, we have already used Neurokernel to successfully scale up the retinal model described in the Integration of Independently Developed LPU Models section to emulate the retina of the house fly, which comprises almost 10 times as many differential equations (18.8 billion) as that of the fruit fly (1.95 billion). Further development of Neurokernel’s support for multiple GPUs and—eventually—neuromorphic hardware will hopefully open the doors to collaborative modeling of the brains of even more complex organisms such as the zebra fish and mouse.

## Future Development

**Fig 10:**
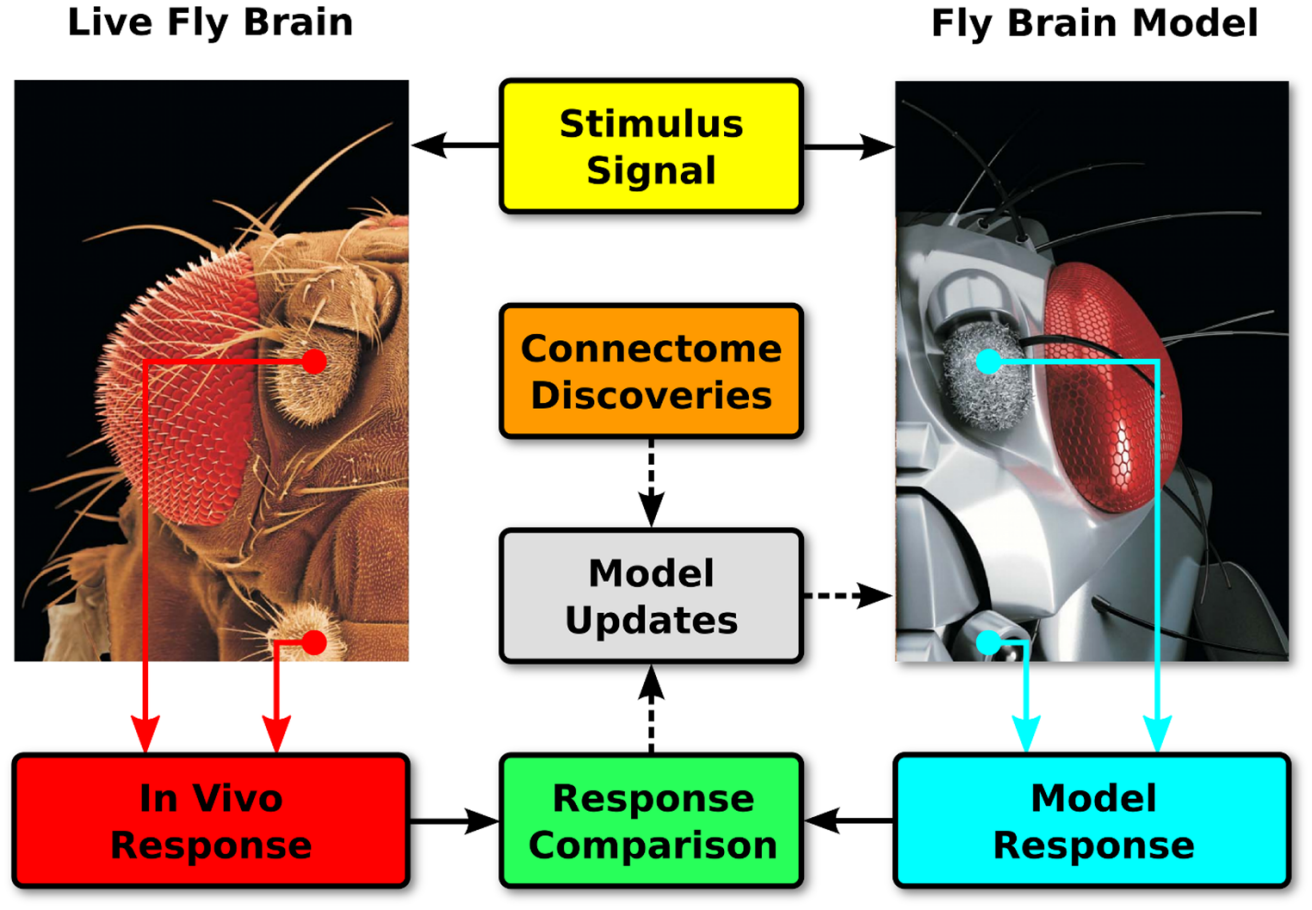
In vivo validation is essential to the development of accurate fly brain models. Neural responses to sensory stimuli are recorded from the live fly brain in real time and compared to the computed responses of the corresponding components in a fly brain model executed on the same time scale. Discrepancies between these responses and new connectome data may be used to improve the model’s accuracy (fruit fly photograph adapted from Berger and fly robot image adapted from Vizcano, Benton, Gerber, and Louis, both reproduced with permission).

Efforts at reverse engineering the brain must ultimately confront the need to validate hypotheses regarding neural information processing against actual biological systems. In order to achieve biological validation of the Neurokernel, the computational modeling of the fruit fly brain must be tightly integrated with increasingly precise electrophysiological techniques and the recorded data evaluated with novel system identification methods [10, 12, 66–70]. This will enable direct comparison of the output of models executed by Neurokernel to that of corresponding neurons in the brain regions of interest. Given that recently designed GPU-based systems for emulating neuronal networks of single spiking neuron types have demonstrated near real-time execution performance for networks of up to ∼10^5^ spiking neurons and ∼10^7^ synapses using single GPUs [30, 33, 71], and in light of advances in the power and accessibility of neuromorphic technology [52, 62–65], we anticipate that future advances in parallel computing technology will enable Neurokernel’s model execution efficiency to advance significantly towards the time scale of the actual fly brain. These advances will enable researchers to validate models of circuits in the live fly’s brain within similar time scales and use the observed discrepancies to inform subsequent model improvements (Fig 10).

Although Neurokernel currently permits brain models to make use of multiple GPUs, it requires programmers to explicitly manage the GPU resources used by a model’s implementation. Having implemented the API for building and interconnecting LPUs described in the Application Programming Interface section within Neurokernel’s application plane, our next major goal is to implement a prototype GPU resource allocation mechanism within the control plane to automate selection and management of available GPUs used to execute a fly brain model. Direct access to GPUs will also be restricted to modeling components implemented by LPU developers and added to Neurokernel’s compute plane; models implemented or defined in the application plane will instantiate and invoke these components. These developments will permit experimentation with different resource allocation policies as LPU models become more complex. Restricting parallel hardware access to modeling components exposed by the compute plane will also facilitate development of future support for other parallel computing technologies such as non-NVIDIA GPUs or neuromorphic hardware.

Neurokernel is a fundamental component of the collaborative workflow needed to accelerate the process of fruit fly brain model development, execution, and refinement by multiple researchers. This workflow, however, also requires a means of efficiently constructing brain models and modifying their structure and parameters in light of output discrepancies observed during validation or to incorporate new experimental data. As noted in the Application Programming Interface section, Neurokernel currently can execute LPU models declaratively specified as GEXF files that each describe an individual LPU’s design as a graph of currently supported neuron and synapse model instances and separately specified inter-LPU connectivity patterns. Since this model representation must either be manually constructed or generated by ad hoc processing of connectome data, modification of LPUs is currently time consuming and significantly slows down the improvement of brain models. LPUs explicitly implemented in Python that do not use supported neuron or synapse models are even less easy to update because of the need to explicitly modify their implementations.

To address these limitations and enable rapid updating and reevaluation of fly brain models, we are building a system based upon graph databases called NeuroArch for the specification and sophisticated manipulation of structural data associated with LPU models and inter-LPU connectivity [72]. NeuroArch will (1) provide LPU developers with a means of defining model components and canonical circuit abstractions using biologically-oriented model-specific labels, (2) enable powerful queries against the data associated with multiple interconnected LPU models via an object-oriented interface similar to that provided by object-relational mapping (ORM) software to web application developers, (3) provide access to model data at different levels of structural abstraction higher than neurons and synapses, (4) enable access to and/or modification of stored data in multiple modes, i.e., as a subgraph (to facilitate graph-based queries) or a table (to facilitate tabular or relational queries), and (5) provide an interface to Neurokernel that enables immediate execution of models defined in NeuroArch.

## Conclusion

Despite the fruit fly brain’s relative numerical tractability, its successful emulation is an ambitious goal that will require the joint efforts of multiple researchers from different disciplines. Neurokernel’s open design, support for widely available commodity parallel computing technology, and ability to integrate independently developed models of the brain’s functional subsystems all facilitate this joining of forces. The framework’s first release is a step in this direction; we expect and anticipate that aspects of the current design such as connectivity structure and module interfaces will be superseded by newer designs informed by the growing body of knowledge regarding the structure and function of the fly brain. We invite the research community to join this effort on Neurokernel’s website (https://neurokernel.github.io/), online code repository (https://github.com/neurokernel/neurokernel), and development mailing list (https://lists.columbia.edu/mailman/listinfo/neurokernel-dev).

## Supporting Information

S1 Video Natural video signal input and photoreceptor/neuron outputs of integrated retina/lamina LPU models. This video depicts a natural video signal input to the photoreceptors in the 721 ommatidia comprised by the retina model, average photoreceptor response per ommatidium, and outputs (membrane potentials) of select photoreceptors (R1) in retina and neurons (L1, L2) in the lamina.
(ZIP)
